# Tazarotene-induced gene 1 inhibits prostaglandin E2-stimulated HCT116 colon cancer cell growth

**DOI:** 10.1186/1423-0127-18-88

**Published:** 2011-11-30

**Authors:** Fu-Ming Tsai, Chang-Chieh Wu, Rong-Yaun Shyu, Chun-Hua Wang, Shun-Yuan Jiang

**Affiliations:** 1 Department of Research, Buddhist Tzu Chi General Hospital Taipei Branch, 289 Jianguo Rd, Sindian District, New Taipei City, 231 Taiwan; 2 Department of Internal Medicine, Buddhist Tzu Chi General Hospital Taipei Branch, 289 Jianguo Rd, Sindian District, New Taipei City, 231 Taiwan; 3 Department of Dermatology, Buddhist Tzu Chi General Hospital Taipei Branch, 289 Jianguo Rd, Sindian District, New Taipei City, 231 Taiwan; 4 Department of Surgery, Tri-Service General Hospital, 325 Chengung Rd, Sec 2, Taipei, 114 Taiwan; 5 School of Medicine, Tzu Chi University, 701 Zhongyang Rd, Sec 3, Hualien, 970 Taiwan

**Keywords:** prostaglandin E2, TIG1, RARRES1, GRK5, β-catenin, colon cancer

## Abstract

**Background:**

The tazarotene-induced gene 1 (*TIG1*) is a putative tumor suppressor gene. We have recently demonstrated both TIG1A and TIG1B isoforms inhibited cell growth and induced the expression of G protein-coupled receptor kinase 5 (GRK5) in colon cancer cells. Because elevated prostaglandin E2 (PGE2) signaling plays a significant role in colorectal carcinogenesis, the objective of this study was to explore the effect of TIG1 on PGE2-induced cellular proliferation and signaling in colon cancer cells.

**Methods:**

HCT116 cells as well as TIG1A and TIG1B stable cells established from HCT116 colon cancer cells using the GeneSwitch system were used. TIG1 isoform expression was induced by mifepristone treatment in stable cells. Cell growth was determined using the WST-1 cell proliferation assay. Activation of β-catenin/TCF and cyclic adenosine monophosphate (cAMP)/CREB signaling pathways were determined using luciferase reporter assays. Expression and subcellular distribution of β-catenin were analyzed using Western blot and confocal microscope. Levels of cAMP were measured using an enzyme immunoassay. RNA interference was used to examine the effects of TIG1- and GRK5-mediated changes.

**Results:**

PGE2-stimulated cell growth was reduced in inducible TIG1A- and TIG1B-stable HCT116 cells. GRK5 expression was upregulated by both TIG1A and TIG1B isoforms, and its expression suppressed PGE2-stimulated HCT116 cell growth. GRK5, TIG1A, and TIG1B expression significantly inhibited PGE2-stimulated β-catenin/TCF and cAMP signaling pathway reporters and cAMP. Also, PGE2-stimulated nuclear localization of β-catenin was inhibited by expression of TIG1A and TIG1B, which was ameliorated by both TIG1 and GRK5 siRNAs.

**Conclusions:**

TIG1 suppressed PGE2-stimulated Wnt and cAMP signaling pathways in colon cancer cells through GRK5.

## Background

The tazarotene-induced gene 1 (*TIG1*) gene, also known as retinoic acid receptor responder 1 (*RARRES1*) gene [1], may be a tumor suppressor [2,3]. Its expression is frequently downregulated through promoter hypermethylation in various carcinomas [3-10]. Ectopic expression of the TIG1A and TIG1B isoforms suppress cellular growth and/or invasion of cancer cells [2,3,5,11]. TIG1 is differentially expressed in spontaneously regressing melanoma [12] and related to cellular differentiation of mesenchymal stem cells [13] and colorectal carcinomas [14]. TIG1 is a carboxypeptidase inhibitor for ATP/GTP binding protein-like 2 (AGBL2) [15].

Prostaglandin E2 (PGE2), which is regulated by cyclooxygenase-2 (COX-2), promotes the growth and invasion of colorectal tumors [16]. PGE2 receptors, which are G protein-coupled receptors (GPCRs), consist of four subtypes, namely EP1-4 [17]. Signaling through EP2 activates the protein kinase A (PKA) pathway that results in phosphorylation of cyclic adenosine monophosphate (cAMP) response element binding protein (CREB) [17]. The Wnt signaling pathway, which is activated in most colorectal cancer cells and some precancerous lesions, is also activated by PGE2 [18,19]. PGE2-stimulated GPCRs stabilize cytosolic β-catenin, resulting in nuclear β-catenin accumulation and transcription factor 7 (TCF7)-mediated transcription [19-22].

G protein-coupled receptor kinases (GRKs) inhibits GPCR signaling through phosphorylation-dependent [23] and -independent mechanisms [24]. The GRK family is comprised of seven members with various tissues distributions. GRK-2, -3, -5, and -6 are expressed ubiquitously [25]. GRKs also bind directly to non-GPCR complexes, such as p38 mitogen-activated protein kinases [26], IκB [27], and p53 [28].

The TIG1A isoform (NP_996846.1) shares the N-terminal 224 amino acids with TIG1B, (NP_002879.2). Expression of both TIG1A and TIG1B isoforms upregulated GRK5 expression and inhibited the growth of HCT116 and SW620 colon cancer cells [11]. GRK5 plays an important role in the TIG1-mediated growth inhibition, since knockdown GRK5 expression significantly alleviated TIG1A-induced growth suppression. PGE2 plays pivotal roles in colorectal carcinogenesis, possibly related activation of the Wnt signaling pathway through the increased nuclear β-catenin [19]. However, whether GRK5 regulates PGE2-mediated growth stimulation has yet to be determined. The objective of the present study was to determine the effects of TIG1 expression on PGE2-mediated cell growth and the β-catenin/TCF and cAMP/CREB signaling pathways, and to investigate the possible role of GRK5 in TIG1-mediated suppressive effects.

## Methods

### Construction of expression vectors

Constitutive expression vectors that encoded myc-tagged TIG1A (pTIG1A-myc) or TIG1B (pTIG1B-myc) fusion proteins have been described previously [11]. Constitutive expression vectors encoding a myc-tagged GRK5 (pGRK5-myc) fusion protein was constructed as follows. The GRK5 cDNA fragment was amplified from human HeLa Tet-off (HtTA) cervical cancer cells, obtained from Dr. T.-C. Chang (Department of Biochemistry, National Defense Medical Center, Taiwan) using GRK5-specific primers (sense 5'-TCGAATTCCATGGAGCTGGAAAACATCGTG-3' and antisense 5'-CGGGATCCGCTGCTTCCGGTGGAG-3'). cDNAs were digested with *Eco*RI and *Bam*HI and subcloned in-frame into the pcDNA3.1-myc-his A expression vector (Invitrogen, Carlsbad, CA, USA).

### Cell culture and transfection

HCT116 colon cancer cells (Food Industry Research and Development Institute) were maintained in growth medium consisting of McCoy's 5A medium (BioWest, Nuaille, France) supplemented with 25 mM HEPES, 26 mM NaHCO_3_, 2 mM L-glutamine, 100 units/mL penicillin, 100 μg/mL streptomycin, and 10% fetal bovine serum (Hyclone, Logan, Utah, USA) at 37°C and 5% CO_2_.

HCT116 or stable cells in six-well plates were transfected with the constitutive expression plasmids described in Wu et al [11] and small interfering RNAs (siRNAs). Plasmids (250 ng of expression plasmid, 250 ng of reporter plasmid, and 30 nM siRNA) and Lipofectamine (GIBCO BRL, Gaithersburg, MD, USA) were diluted in Opti-MEM medium (GIBCO BRL). The DNA/Lipofectamine complexes were added to cells and incubated for 2.5 h at 37°C. The cells were refreshed with complete medium and were incubated for 24 h at 37°C for further analysis.

### RNA interference

Three TIG1 siRNAs (Ambion, Austin, TX, USA), which targeted both TIG1A and TIG1B, were synthesized and targeted the nucleotides 488 to 508, 540 to 560, and 596 to 616, based on Genbank accession NM_206963. GRK5 siRNAs were targeted to nucleotides 452 to 472, 2158 to 2178, and 2406 to 2426 according to Genbank accession NM_005308. The Silencer^® ^negative control #1 siRNA (Ambion) was used as a negative control. Inducible TIG1A or control stable HCT116 cells were cultured for 24 h in 6-well plates prior to transfection.

### Inducible TIG1 stable clones

Inducible TIG1A, TIG1B, and control stable clones were established from HCT116 cells using the GeneSwitch system [29] with minor modification as described previously [11]. TIG1A and TIG1B, expressed as V5-tagged recombinant proteins, in stable cells were induced followed by exposure to the synthetic progestin mifepristone (MFP). MFP actives the recombinant regulatory protein, which contains a DNA binding domain from the yeast GAL4 protein, a ligand binding domain from the progesterone receptor, and an activation domain from the NF-κB protein [30]. Stable clones were maintained in growth medium containing hygromycin and zeocin and switched to medium without hygromycin and zeocin overnight prior to incubation with MFP.

### Cellular proliferation analysis

Control and TIG1-inducible HCT116 cells (2 × 10^4^) were seeded overnight and then washed twice with phosphate buffered saline (PBS, 3.2 mM Na_2_HPO_4_, 0.5 mM KH_2_PO_4_, 1.3 mM KCl, 135 mM NaCl, pH 7.4). Cells were incubated with serum-free medium for 16 h, and then cultured in serum-free medium containing MFP (5 nM) or ethanol (0.1%) vehicle alone or in combination with or without PGE2 (10 nM) for 48 h. WST-1 reagent (100 μL; Roche, Germany) was added for 4 h at 37°C. Absorbance at 450 and 650 nm was recorded. The percentage of cell growth relative to control cells was defined as [(A_450_-A_650_) of MFP or PGE2-treated cells/(A_450_-A_650_) of vehicle cells] × 100%. All experiments were performed in triplicate.

To investigate the effects of GRK5 on PGE2-stimulated cell growth, HCT116 cells were plated overnight and then transfected with 150 ng of GRK5 or control expression vector for 24 h. Cells were washed twice with PBS, incubated in serum-free growth medium for 16 h and then cultured in the absence or presence of 10 nM PGE2 for 48 h. Cell proliferation was measured using the WST-1 reagent as described above.

### Western blot analysis

Total cytosol extracts were prepared in lysis buffer (25 mM HEPES, pH 7.5, 150 mM NaCl, 1% Igepal CA-630, 10 mM MgCl_2_, 1 mM EDTA, and 10% glycerol) containing 20 μg/mL aprotinin and 20 μg/mL phenylmethylsulfonyl fluoride and 2 mM NaF and 1 mM Na_3_VO_4_. Proteins (5 to 100 μg) were separated using 10-12% polyacrylamide gels containing sodium dodecyl sulfate and were then transferred to polyvinylidene difluoride membranes. After blocking, the membranes were incubated with anti-V5 (Invitrogen), anti-myc (Invitrogen), anti-GRK5 (Santa Cruz Biotechnology, Santa Cruz, CA, USA), anti-β-catenin (Sigma, Saint Louis, MO, USA), anti-lamin B1 (Invitrogen), anti-glyceraldehyde 3-phosphate dehydrogenase (GAPDH; Cell Signaling Technology, Beverly, MA, USA), or anti-actin (Sigma) antibodies for 12 h at 4°C. The appropriate horseradish peroxidase-conjugated secondary antibodies (Calbiochem, Darmstadt, Germany) was then added at room temperature for 1 h. Specific protein bands were developed using the Amersham ECL (Amersham, Bucks, UK). Relative protein expression was quantified following normalization to the housekeeping gene protein levels.

### Nuclear and cytoplasmic fractionation

To determine the nuclear or cytosolic distribution of β-catenin, cell fractionation was performed using the Qproteome cell compartment kit (Qiagen, Valencia, CA, USA) according to the manufacturer's instructions with the addition of protease and phosphatase inhibitors to all buffers. Nuclear (10 μg) and cytosolic (25 μg) extracts were analyzed using Western blot analysis. Cytosolic β-catenin levels were first normalized to GAPDH levels, and nuclear β-catenin levels were normalized to lamin B1 levels. Cytosolic β-catenin was then normalized to cytosolic β-catenin protein levels in the respective cells or control stable cells without PGE2 treatment. Similarly, nuclear β-catenin levels were then normalized to nuclear β-catenin protein levels in the respective cells or control stable cells without PGE2 treatment for the siRNA study. Finally, relative levels of nuclear β-catenin relative to that of cytosolic β-catenin were calculated, and the level of nuclear β-catenin in the respective cells or in control stable cells without PGE2-treatment was designated as 1.0.

### Luciferase reporter assay

TOPFLASH (Upstate Biotechnology, Lake Placid, NY, USA) and CREB reporter (pCREB-Luc; Stratagene, La Jolla, CA, USA) activities were assessed in transiently transfected HCT116 cells. HCT116 cells (1 × 10^5^) were plated in six-well plates in growth medium for 24 h. After transient transfection with 500 ng plasmids, the cells were serum starved for 16 h and stimulated with 10 nM PGE2 for 24 h at 37°C. Alternatively, inducible TIG1A, TIG1B, or control stable cells were plated overnight, transfected with 500 ng reporter plasmid, incubated with 5 nM MFP for 24 h, serum starved for 16 h, and incubated with PGE2 (10 nM) for 24 h. To analyze the effect of GRK5 or TIG1 knockdown on reporter activity, inducible or control stable cells were cotransfected with siRNAs and reporter plasmids followed by serum starvation and then stimulation with 5 nM MFP for 24 h. MFP was present during the subsequent serum starvation and PGE2 treatment.

Cells were harvested in reporter lysis buffer (Promega, Madison, WI, USA). Luciferase activity was measured using the luciferase assay kit (Stratagene) and a multi-functional microplate reader (Infinite F200, Tecon, Durham, NC, USA). Relative luciferase activity of each sample was determined after normalizing for the protein concentration of each lysate. All experiments were performed in triplicate.

### Confocal and immunofluorescent analysis

Stable cells (1 × 10^5^) were plated on poly-L-lysine-coated coverslips in 35-mm dishes in growth medium. Cells were treated with 5 nM MFP for 24 h, maintained in serum-free medium for 16 h, treated with 10 nM PGE2 for 30 min, washed twice with PBS, and fixed with 4% paraformaldehyde. Alternatively, cells were transfected with siRNAs prior to MFP treatment, serum starvation, and PGE2 treatment. MFP was present during the serum starvation and PGE2 treatment. Cells were permeabilized in 0.1% Triton X-100/PBS for 15 min. After washing, cells were blocked in PBS containing 2% bovine serum albumin for 1 h at room temperature, incubated with anti-β-catenin antibody at 37°C for 2 h followed by the Alexa Fluor^@ ^488 goat anti-mouse IgG for 1 h at room temperature, stained with 1 μg/mL 4'6-diamidino-2-phenylindole (DAPI), and analyzed with a laser scanning confocal microscope (Leica TCS SP5 scanner, Leica, Bensheim, Germany). Percentage of cells expressing nuclear β-catenin was determined by randomly analyzing 250 to 300 cells from each treatment group.

### Measurement of cAMP levels

Cells were cultured onto 6-well plates overnight and then incubated with 5 nM MFP in complete medium for 24 h. After washing twice with PBS, cells were serum starved for 16 h and then incubated in the absence or presence of 10 nM PGE2 for 5 min. Cells were lysed with 0. 1 N HCl for 20 min, scraped, collected by centrifugation. Levels of cAMP in the supernatants were determined using a cyclic AMP EIA kit (Cayman Chemical, Ann Arbor, MI, USA) according the manufacturer's instructions.

### Statistical analysis

Numerical data are shown as mean ± standard deviation (SD) of triplicates from each independent experiment. Cell proliferation and luciferase activities were analyzed using the student's *t*-test. *P*-values less than 0.05 were considered statistically significant.

## Results

### TIG1A and TIG1B upregulate GRK5 expression and suppress PGE2-stimulated HCT116 cell growth

**Figure 1:**
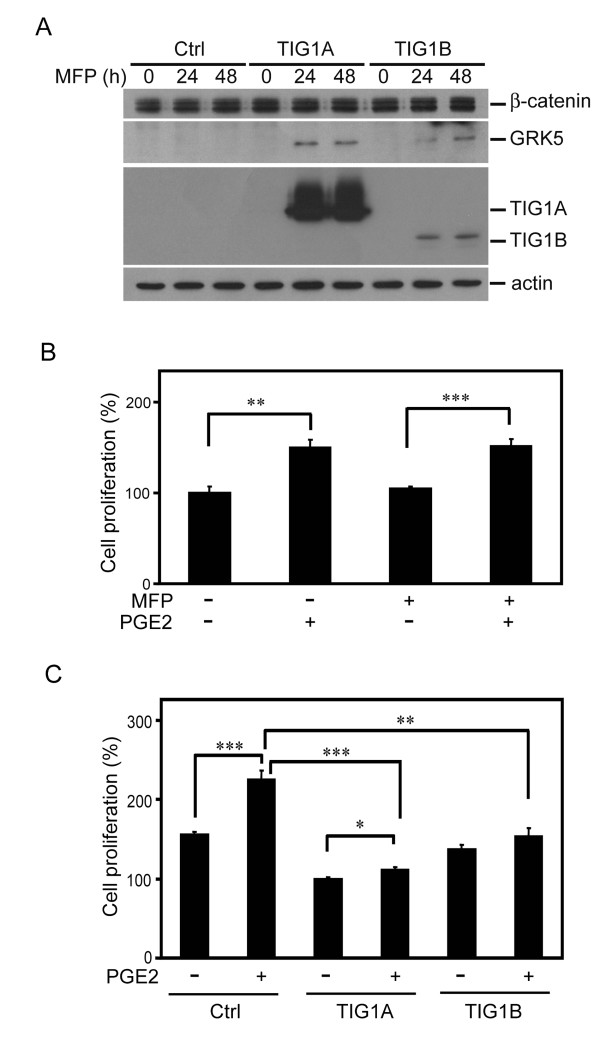
**Effects of TIG1A and TIG1B expression on PGE2-stimulated HCT116 cell proliferation**. (A) Western blot analysis of TIG1A, TIG1B, GRK5 and β-catenin expression in MFP (5 nM)-induced TIG1 and control (Ctrl) stable clones. (B) Effect of MFP on PGE2-stimulated growth of control stable cells. (C) Effects of TIG1A and TIG1B expression on PGE2-induced HCT116 cell growth. Representative results of three independent experiments are shown. *, *P *< 0.05; **, *P *< 0.01; ***, *P *< 0.001.

In the COX-2-negative HCT116 cells, TIG1A and TIG1B expression was markedly induced after the respective stable cells were incubated with 5 nM MFP for 24 and 48 h (Figure 1A). GRK5 expression was evidently increased in both TIG1A and TIG1B stable cells exposed to 5 nM MFP for 24 to 48 h. No basal TIG1A, TIG1B and GRK5 expression was detected in MFP-treated control stable cells as well as TIG1A and TIG1B stable cells in the absence of MFP treatment. In addition, the expression of β-catenin was not altered. "Stable TIG1A- or TIG1B-expressing cells" were used to denote inducible TIG1A or TIG1B cells exposed to MFP (5 nM) treatment thereafter.

In control HCT116 stable cells, PGE2 (10 nM) treatment for 48 h significantly stimulated cell proliferation, and the effect was not altered by co-incubation with 5 nM MFP (Figure 1B). However, levels of PGE2-stimulated growth in stable TIG1A- and TIG1B-expressing cells were significantly suppressed (Figure 1C). Therefore, MFP induced TIG1A, TIG1B and GRK5 expression in the inducible HCT116 stable cells. Also, PGE2-stimulated growth was significantly suppressed by induced expression of TIG1A and TIG1B. A similar inhibition of PGE2-stimulated growth was also demonstrated in inducible TIG1A, TIG1B and control stable cells established from COX-2-positive SW620 colon cancer cells [11,31] (additional file 1).

### TIG1 suppressed PGE2-stimulated TOPFLASH and CREB reporter activities and β-catenin nuclear localization

**Figure 2:**
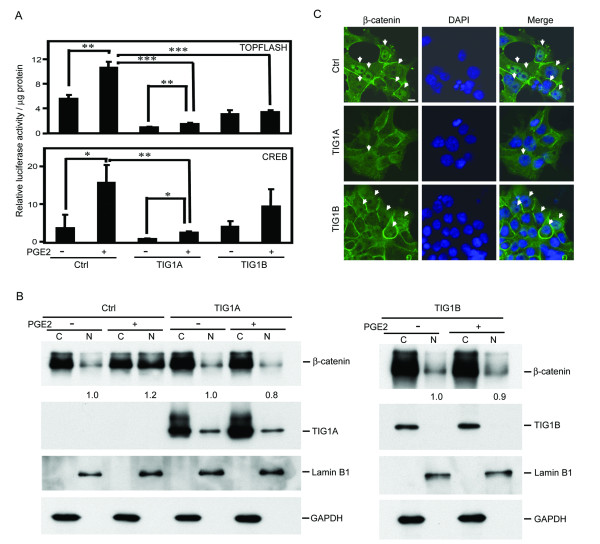
**Effects of TIG1A and TIG1B expression on β-catenin/TCF- and CREB-mediated activities and β-catenin subcellular localization on TIG1A- and TIG1B-expressing HCT116 cells**. **(**A) β-catenin/TCF (top panel)- and CREB (bottom panel)-mediated activities were analyzed using luciferase reporters. Indicated stable cells were transfected with the reporter plasmid and then incubated with 5 nM MFP for 24 h, serum starved for 16 h, and then treated with or without PGE2 (10 nM) for 24 h. MFP was present during serum starvation and PGE2 treatment in all similar experiments. Cellular lysates were analyzed for transactivation activity of the TOPFLASH or CREB luciferase reporters. Results were expressed as means ± SD from triplicate samples after normalization to protein concentration and then to the results obtained from TIG1A-inducible cells without PGE2 treatment. *, *P *< 0.05; **, *P *< 0.01; ***, *P *< 0.001. (B) Effects of TIG1 isoforms on β-catenin subcellular distribution were determined by subcellular fractionation and Western blot analysis. Indicated stable cells were incubated with 5 nM MFP for 24 h, serum starved for 16 h, and incubated with or without 10 nM PGE2 for 30 min. After subcellular fractionation, β-catenin, TIG1A, TIG1B, lamin B1, and GAPDH levels were determined by Western blot analysis. Normalization of nuclear (N) and cytosolic (C) β-catenin levels was described in the Materials and Methods section, and the respective cells without PGE2-treatment were designated as 1.0. (C) Effects of TIG1 isoform expression on β-catenin subcellular distribution was determined by immunofluorescent analysis. Stable cells were incubated with MFP, serum starved, and incubated with PGE2 for 30 min as described above. β-catenin localization (green) and nuclei (blue) were analyzed using a laser scanning confocal microscope. Bars, 10 μm. Arrows indicate nuclear β-catenin.

TOPFLASH and CREB reporter assays were first used to investigate the effects of TIG1 expression on PGE2-stimulated Wnt and PKA signaling pathways by measuring β-catenin/TCF- and cAMP/CREB-mediated reporter activities, respectively. PGE2 treatment significantly stimulated TOPFLASH and CREB reporter activities in control HCT116 stable cells (Figure 2A). However, in stable TIG1A- and TIG1B-expressing cells, PGE2-stimulated TOPFLASH reporter activity was significantly decreased, and PGE2-stimulated CREB reporter activity was significantly suppressed in TIG1A-expressing cells.

The effects of TIG1 on PGE2-stimulated β-catenin nuclear localization in HCT116 cells were then determined by Western blot analysis of cytosolic and nuclear extracts. After exposure to 10 nM PGE2 for 5 min to 1 h, the greatest nuclear β-catenin accumulation as determined by immunofluorescent analysis was evident at 30 min in control stable cells (data not shown). The majority of TIG1 isoforms were detected in the cytosolic fractions (Figure 2B). Levels of nuclear β-catenin in MFP-treated control stable cells were increased to 1.2-fold followed by exposure to PGE2 (10 nM) for 30 min. However, PGE2 did not stimulate nuclear β-catenin accumulation in both TIG1A- and TIG1B-expressing cells. Similar results were obtained using immunofluorescent staining of β-catenin in response to PGE2 stimulation in MFP-treated stable cells (Figure 2C). In control HCT116 stable cells, nuclear β-catenin was detected in 5.4% of vehicle-treated cells (data not shown), which increased to 31.5% upon PGE2 stimulation (top panel). However, PGE2 treatment of stable TIG1A- and TIG1B-expressing cells resulted in nuclear β-catenin detected in only 8.7 and 11.0% of cells, respectively. In addition, the intensity of nuclear β-catenin staining in PGE2-stimulated TIG1A and TIG1B expressing cells was much weaker than that observed in PGE2-and MFP-treated control stable cells.

Thus, upon induced expression of both TIG1A and TIG1B, suppression of both PGE2-stimulated TOPFLASH reporter activities and nuclear β-catenin localization in MFP-treated stable HCT116 cells was observed. PGE2-stimulated CREB reporter activities were also suppressed by TIG1B, but to a lesser extent.

### Effects of GRK5 expression on PGE2-stimulated cell growth and TOPFLASH and CREB reporter activities

**Figure 3:**
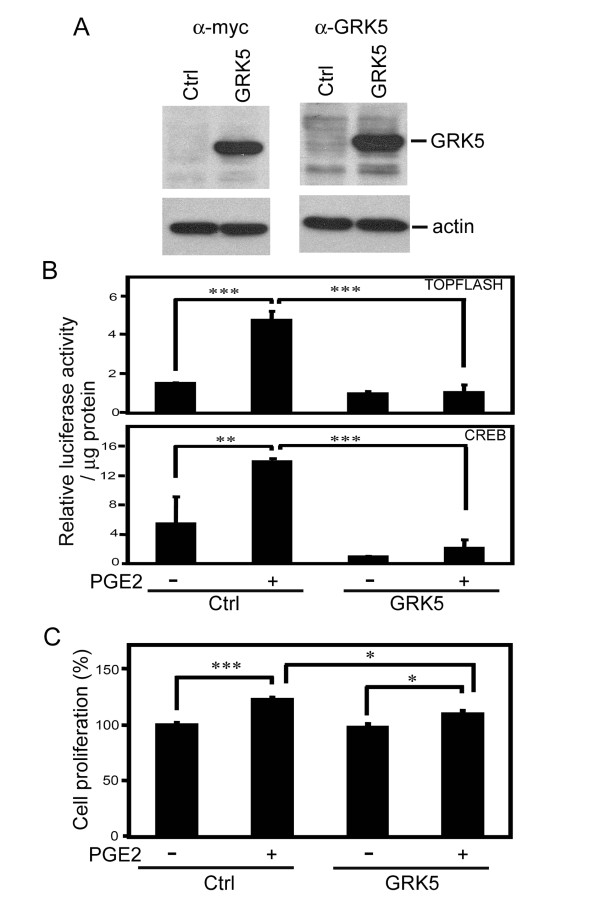
**Effects of GRK5 expression on PGE2-mediated TOPFLASH and CREB reporter activities and cell growth**. (A) HCT116 cells were transfected with control or GRK5 expression vector for 24 h, and expression of the GRK5 fusion protein was determined by Western blot analysis using anti-myc or anti-GRK5 antibodies. Actin expression was used as a loading control. (B) TOPFLASH and CREB reporter activities were determined in HCT116 cells after transient expression of indicated expression vectors for 24 h, serum starvation for 16 h and then PGE2 (10 nM) incubation for 24 h. (C) Cell proliferation was determined in HCT116 cells transfected with control or GRK5 expression vector for 24 h, serum starvation for 16 h, and PGE2 stimulation for 48 h. Representative results from triplicate samples were expressed as means ± SD. *, *P *< 0.05; **, *P *< 0.01; ***, *P *< 0.001. Control, Ctrl.

To investigate the effects of GRK5 on PGE2-mediated signal pathways, GRK5 expression vector was constructed. Expression of the GRK5 fusion protein with an expected molecular weight of 67 kDa was observed in HCT116 cells (Figure 3A). In vector control-transfected cells, PGE2 significantly increased TOPFLASH and CREB reporter activities by 3.1- and 2.5-fold, respectively (Figure 3B), which was not observed in GRK5-expressing HCT116 cells. PGE2-stimulated TOPFLASH and CREB reporter activities were significantly suppressed by 77.4 and 84.4% in GRK5-expressing cells treated with PGE2 (Figure 3B). Because PGE2 stimulates TOPFLASH and CREB reporter activities through the PGE2 receptor subtype 2, EP2, in DLD-1 colon cancer cells [19], the effects of GRK5 expression on PGE2-stimulated TOPFLASH and CREB reporter activities in EP2-transfected HCT116 cells were analyzed. GRK5 suppressed both reporter activities in a dose-dependent manner (additional file 2). Finally, PGE2 treatment for 2 days significantly increased cell growth by 25% in control transfected cells, which was significantly decreased to 11.9% upon GRK5 expression (Figure 3C). Results from transiently transfected HCT116 cells demonstrated that GRK5 suppressed PGE2-stimulated growth as well as TOPFLASH and CREB reporter activities. Also, GRK5 suppressed PGE2-stimulated activation of TOPFLASH and CREB reporters in EP2-transfected cells.

### Effects of TIG1 and GRK5 siRNAs on suppression of PGE2-stimulated TOPFLASH reporter activity and β-catenin nuclear localization

**Figure 4:**
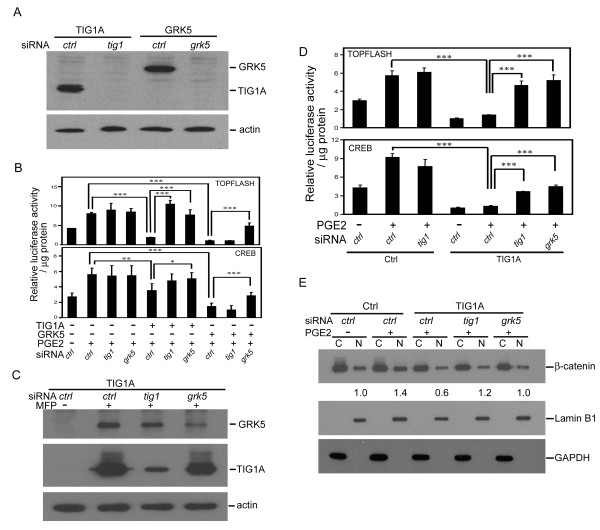
**Effects of TIG1 and GRK5 siRNAs on TIG1-mediated suppression of reporter activities and nuclear β-catenin accumulation**. (A) Western blot analysis of HCT116 cell lysates transiently transfected with constitutive TIG1A or GRK5 expression vectors along with the indicated siRNAs for 48 h using anti-myc antibodies. β-actin expression was determined as a loading control. (B) Effects of TIG1 and GRK5 knockdown on PGE2-stimulated TOPFLASH and CREB reporter activities in transiently transfected HCT116 cells. Cells were cotransfected with indicated TIG1A, GRK5 or control expression vector, reporter plasmid and siRNA for 24 h, serum starved for 16 h, and then incubated with 10 nM PGE2 for 24 h. TOPFLASH (top panel) and CREB (bottom panel) reporter activities were determined. Representative results from triplicate samples were expressed as means ± SD. (C) Effects of TIG1 and GRK5 siRNAs on TIG1A and GRK5 expression in stable TIG1A-expressing cells. Cells were transfected with the indicated siRNAs and then incubated with or without 5 nM MFP immediately after transfection for 24 h. Expression of TIG1A and GRK5 was determined by Western blot analysis using anti-V5 and anti-GRK5 antibodies, respectively. (D) Effects of TIG1A or GRK5 knockdown on PGE2-stimulated TOPFLASH and CREB reporter activities in stable control and TIG1A expressing cells. Cells were transfected with the indicated siRNAs and then incubated with 5 nM MFP for 24 h, serum starved for 16 h, and stimulated with or without 10 nM PGE2 for 24 h. MFP was present during serum starvation and PGE2 treatment. TOPFLASH (top panel) and CREB (bottom panel) reporter activities were analyzed. (E) Effects of TIG1 and GRK5 siRNAs on TIG1A-mediated suppression of PGE2-stimulated nuclear β-catenin accumulation in stable TIG1A-expressing cells. Control and TIG1A stable cells were transfected with the indicated siRNA and then incubated with 5 nM MFP for 24 h, serum starved for 16 h, and treated with or without 10 nM PGE2 for 30 min. MFP was present during serum starvation and PGE2 treatment. Nuclear and cytosolic fractions were prepared, and subcellular distribution of nuclear and cytosolic β-catenin was determined by Western blot analysis. Normalization in the levels of nuclear and cytosolic β-catenin was described in the Materials and Methods, and the relative levels of nuclear β-catenin in control stable cells without PGE2-treatment was designated as 1.0. *, *P *< 0.05; **, *P *< 0.01; ***, *P *< 0.001.

As shown in Figure 4A, expression of TIG1A and GRK5 was completely suppressed when cotransfected with the respective siRNAs but not the control siRNA. Cotransfection of TIG1A or GRK5 expression vector with control siRNA significantly suppressed PGE2-stimulated TOPFLASH reporter activity by 77.1 and 87.5%, respectively (Figure 4B, top panel), which was completely recovered when TIG1A-transfected cells were cotransfected with TIG1 or GRK5 siRNAs. Although both TIG1 and GRK5 siRNAs reduced TIG1-mediated suppression of TOPFLASH reporter activity, only GRK5 siRNAs partially alleviated GRK5-mediated suppression of the reporter activity. PGE2-stimulated CREB reporter activity was significantly suppressed by 37.3 and 75.5% upon transient transfection of TIG1A and GRK5 expression vector, respectively, which was significantly alleviated when cotransfected with GRK5, but not TIG1 siRNAs (Figure 4B, bottom panel).

Similar experiments in stable TIG1A expressing cells revealed a partial knockdown of TIG1A (65.1%) and GRK5 (50%) protein expression using the respective siRNAs (Figure 4C). Levels of PGE2-stimulated TOPFLASH and CREB reporter activities in control siRNA-transfected TIG1A-expressing cells were significantly lower than that of control stable cells (Figure 4D). In stable TIG1A-expressing cells transfected with control siRNA, PGE2 only weakly increased the TOPFLASH and CREB reporter activities. Further, significantly enhanced TOPFLASH and CREB transactivation, ranging from 179 to 271%, was observed upon transfection with TIG1 or GRK5 siRNAs.

The effects of GRK5 and TIG1 knockdown on PGE2-stimulated β-catenin nuclear localization were evaluated in MFP-treated stable cells. In MFP-treated control stable cells transfected with control siRNA, levels of nuclear β-catenin were designated as 1.0-fold in the absence of PGE2 treatment; treatment with 10 nM PGE2 for 30 min increased nuclear β-catenin to 1.4-fold (Figure 4E). In stable TIG1A-expressing cells transfected with control siRNA and treated with PGE2, nuclear β-catenin decreased to 0.6-fold. Upon transfection with siRNA specific for TIG1 or GRK5, nuclear β-catenin increased to 1.2- and 1.0-fold, respectively. Confocal microscopic analysis also confirmed that both TIG1 and GRK5 siRNAs alleviated the suppression of PGE2-stimulated nuclear β-catenin in TIG1A-expressing cells (additional file 3). Basal levels of nuclear β-catenin in TIG1A-expressing cells were analyzed in TIG1A-expressing cells transfected with control siRNA and then incubated with or without 10 nM PGE2 for 30 min. In contrast to the increase in PGE2-stimulated nuclear β-catenin observed in control stable cells transfected with control siRNA, PGE2 treatment did not increase nuclear β-catenin in control siRNA transfected TIG1A-expressing cells (additional file 4).

Using siRNA, TIG1A-mediated suppression of TOPFLASH reporter activity and nuclear β-catenin localization was alleviated by both TIG1 and GRK5 siRNAs, whereas GRK5-mediated suppression of TOPFLASH reporter activity was reversed by GRK5, but not TIG1 siRNA. Similar, but less potent, effects were observed for CREB reporter activity.

### Effects of TIG1 on cAMP levels

**Figure 5:**
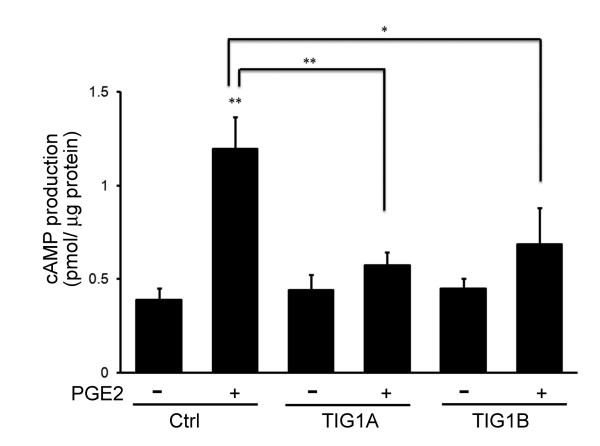
**Effects of TIG1 isoforms on cAMP levels**. Control (Ctrl), TIG1A and TIG1B stable HCT116 cells were incubated with 5 nM MFP for 24 h, serum starved for 16 h, and then treated with or without PGE2 (10 nM) for 5 min. Levels of cAMP were measured using an enzyme immunoassay. Representative results from triplicate samples were expressed as mean ± SD. *, *P *< 0.05; **, *P *< 0.01.

The effects of TIG1 on PGE2-stimulated cAMP/PKA signaling were analyzed by measuring the levels of cAMP. PGE2 stimulation for 5 min significantly increased cAMP levels in control stable HCT116 cells, but not in TIG1A- or TIG1B-expressing cells. Levels of PGE2-stimulated cAMP were significantly decreased by 52.2 and 42.8% in TIG1A- and TIG1B-expressing cells, respectively (Figure 5).

## Discussion

The TIG1A and TIG1B isoforms exhibit growth inhibitory activities on several cancer cells, including HCT116 and SW620 colon cancer cells [2,3,5,11]. The growth suppressive activities of TIG1 may be related to the regulation of cellular apoptosis and cell cycle progression. This is supported by our recent observation of increased lactate dehydrogenase release in TIG1B-transfected HCT116 cells, differential expression of apoptosis-related genes in TIG1A- and TIG1B-expressing cells, and delayed S phase progression in stable TIG1A-expressing HCT116 cells [11]. The effects of TIG1 on the regulation of cell cycle are further supported by the regulation of tubulin phosphorylation cycles through inhibiting the activity of carboxypeptidase AGBL2 [15]. The present study further demonstrates that both TIG1 isoforms inhibited PGE2-stimulated growth, TOPFLASH and CREB reporter activities, cAMP levels and β-catenin nuclear translocation.

PGE2 promotes cell growth and angiogenesis, through activation of EP GPCRs [16]. In DLD-1 and LS174T colon cancer cells, activation of the EP2 receptor by PGE2 promotes the release of glycogen synthase kinase 3β (GSK-3β) from the adenomatous polyposis coli (APC)/Axin/β-catenin complex [19]. PGE2 also increases GSK-3β phosphorylation through PI3K/AKT [19] and protein kinase A signaling [22]. Subsequent suppression of β-catenin degradation results in increased nuclear β-catenin accumulation and expression of genes associated with cell growth and invasion, such as cyclin D1 and vascular endothelial growth factor.

HCT116 cells harbor a somatic β-catenin mutation [32]; the serine deletion mutation renders it resistant to GSK-3β-induced degradation [33], which is consistent with the high levels of cytoplasmic β-catenin observed before and after PGE2 treatment in the present study. However, the significance of β-catenin mutations in colorectal carcinogenesis remains to be fully elucidated; no differences in growth rate or *in vitro *anchorage-independent growth have been observed [34]. Regardless, increased nuclear β-catenin accumulation and subsequent TOPFLASH reporter activity following PGE2 treatment was observed in the present study, suggesting that PGE2 induced β-catenin transactivation rather than inhibited its degradation in HCT116 cells. Thus, TIG1 isoforms might suppress PGE2-stimulated cell proliferation through inhibition of the β-catenin/TCF transactivation. Alternatively, because TIG1 isoforms also suppressed PGE2-induced cAMP levels and cAMP/CREB reporter activities, they might directly or indirectly affect the PGE2 receptor or downstream signaling pathways that lead to growth inhibition [35]. Since both intracellular signaling pathways were inhibited by TIG1, the mechanism by which TIG1 inhibited PGE2-stimulated growth is therefore likely to take place at the level involving EP receptor activation.

Studies analyzing the effect of GRKs on EP receptor activation or PGE2-stimulated activities are limited. GRK2, GRK3, and GRK5 mediate PGE2-stimulated phosphorylation and desensitization of EP4 [36]. GRK5 downregulates ligand-induced GPCR signaling through internalization [37]. Our previous study demonstrated an important role of GRK5 in TIG1A-mediated growth inhibition of stable TIG1A-expressing cells [11]. This study further demonstrated that PGE2-stimulated TOPFLASH and CREB reporter activities were downregulated by TIG1, and the effects were alleviated by GRK5 siRNA. Therefore, TIG1 may increase GRK5 expression, which subsequently desensitizes the PGE2 receptor through receptor phosphorylation, thereby downregulating β-catenin/TCF/TOFFLASH- and CREB-mediated transactivation. EP2 has been shown to mediate PGE2-stimulated β-catenin/TCF and cAMP/CREB pathways [19]. Through transient transfection, this study demonstrated that GRK5 dose-dependently suppressed PGE2-stimulated TOPFLASH and CREB reporter activities in EP2-transfected HCT116 (additional file 2) and DLD-1 (data not shown) cells. Therefore, EP2 may be one of the targeted receptor desensitized by GRK5. We were unable to detect endogenous EP2 expression using Western blot analysis. Whether the TIG1-induced and GRK5-mediated inhibition of PGE2-stimulated signaling in HCT116 cells is mediated through EP2 or other receptors remains to be elucidated.

GRK5 also functions through non-GPCR pathways, phosphorylating the low density lipoprotein receptor-related protein 6 (LRP6) and stimulating Wnt/LRP6 signaling [38]. Therefore, the observed inhibitory effects of GRK5 on PGE2-stimulated reporter activities may represent the net result after balancing the stimulatory signal induced by GRK5 on LRP6 phosphorylation. Also, GRK5 binds and regulates p53 [28] and IκBα [27]; whether they play a role in GRK5- and TIG1-mediated inhibition of PGE2-stimulated activities merits further investigation. Results shown in this and our recent study [11] have demonstrated that GRK5, TIG1A, or TIG1B alone exhibited growth-suppressive activities. The variation in the levels of GRK5 and the extent of inhibition in PGE2-stimulated growth between TIG1A- and TIG1-B expressing cells was much less than the difference in levels of MFP-stimulated TIG1A and TIG1B protein expression HCT116 cells as shown in Figure 1. Therefore, GRK5, rather than TIG1 protein, may play a major role in the suppression of PGE2-stimulated HCT116 cell growth.

There are several study limitations that warrant consideration. Firstly, HCT116 cells harbor a somatic β-catenin mutation rendering it resistant to GSK-3β-dependent phosphorylation and degradation [32,33]. Nevertheless, PGE2-mediated nuclear accumulation of β-catenin was observed in the present study, which was altered upon TIG1 expression. Secondly, although it is well known that the COX-2/PGE2/EP2 response pathway plays a critical role in colorectal cancer progression [16], HCT116 cells are COX-negative and, therefore, do not produce endogenous PGE2 [39]. Thus, the effects of TIG1 expression on cell proliferation and β-catenin subcellular localization and transactivation by endogenously produced PGE2 could not be assessed. However, lack of endogenous PGE2 production by HCT116 cells provides the advantage of analyzing the effect of exogenous PGE2 alone. Using SW620 cells that express COX-2 and wild type β-catenin, we observed that cellular growth stimulated by exogenous PGE2 was inhibited by both TIG1 isoforms (additional file 1). In the absence of PGE2 addition, both TIG1 isoforms inhibited the growth of SW620 cells that can produce PGE2 [11]. Similarly, basal cell growth in TIG1A-expressing SW620 stable cells incubated in serum-free medium without PGE2 treatment was significantly lower than that of control stable cells (additional file 1). Considering that TIG1-mediated inhibition of PGE2-stimulated TOPFLASH and CREB reporter activities and nuclear β-catenin accumulation was suppressed by GRK5 siRNAs in HCT116 cells, TIG1 may therefore inhibit SW620 cell growth through inhibition of endogenous PGE2 signaling. Finally, TIG1 is a putative tumor suppressor that is frequently hypermethylated in several cancer tissues; hypermethylation is strongly associated with loss of *TIG1 *gene expression [8,9]. Loss of TIG1 expression [14] and an increase in COX-2 expression [40] are related to the progression of colorectal cancer. Future analysis in reactivation of TIG1 expression in TIG1 hypermethylated cells that do not harbor β-catenin mutation, knockdown of TIG1 expression in high TIG1-expressing cells as well as analysis of the clinical significance between TIG1 and COX-2 expression may provide further information on the physiological significance of TIG1 on PGE2-mediated colorectal carcinogenesis.

## Conclusions

In conclusion, both TIG1A and TIG1B suppressed PGE2-stimulated colon cancer cell proliferation, β-catenin nuclear localization, β-catenin/TCF transactivation, and cAMP/CREB signaling through expression of GRK5. The suppression of β-catenin/TCF transactivation and β-catenin nuclear localization was mediated through GRK5. The results suggest an inhibitory role of TIG1 on PGE2-mediated colorectal carcinogenesis at least in part, through upregulation of GRK5 expression.

## Abbreviations

APC: adenomatous polyposis coli; AGBL2: ATP/GTP binding protein-like 2; CREB: cAMP response element binding protein; CRE: cAMP response element; cAMP: cyclic adenosine monophosphate; COX-2: cyclooxygenase-2; DAPI: 4'6-diamidino-2-phenylindole; GPCR: G protein-coupled receptor; GRKs: G protein-coupled receptor kinases; GSK-3β: Glycogen synthase kinase 3β; GAPDH: glyceraldehyde 3-phosphate dehydrogenase; LRP6: lipoprotein receptor-related protein 6; MFP: mifepristone; PI3K: phosphatidylinositol 3-kinase; PBS: phosphate buffered saline; PGE2: prostaglandin E2; RARRES1: retinoic acid receptor responder 1; siRNA: small interfering RNAs; SD: standard deviation; TIG1: tazarotene-induced gene 1; TCF-7: transcription factor 7.

## Competing interests

The authors declare that they have no competing interests.

## Authors' contributions

F-MT performed the majority of the experiments, contributed to the experimental design, and drafted the manuscript. C-CW, R-YS and C-HW contributed to experimental design and data discussion. S-YJ designed and supervised the experiments, assisted in the writing of and proofed the manuscript. All authors read and approved the final draft of the manuscript.

## Supplementary Material

Additional file 1 **Effects of TIG1A and TIG1B expression on PGE2-induced SW620 cell growth**. Control, TIG1A and TIG1B inducible stable cells established from SW620 colon cancer cells using the GeneSwitch system [11] were plated overnight, serum starved for 16 h and then incubated in serum- free medium in the absence or presence of PGE2 (10 nM) for 48 to 72 h. MFP was present during serum starvation and PGE2 addition. Cell growth was determined using the WST-1 cell proliferation assay. *, *P *< 0.05. Click here for file

Additional file 2 **GRK5 suppressed PGE2-stimulated TOPFLESH and CREB reporter activities in EP2-transfected HCT116 cells**. HCT116 cells were transiently transfected with 250 ng of TOPFLASH or CREB reporter plasmid along with 100 ng of indicated control vector or EP2 TrucClone™ cDNA (PTGER2, OriGene Technologies, Inc. Rockville, MD, USA) and 50 to 150 ng of GRK5 expression vectors for 24 h. Cells were serum starved for 16 h and then incubated with or without 10 nM PGE2 for 24 h. Reporter activities were measured as described in the Materials and Methods. Representative results were expressed as means ± SD from triplicate samples. *, *P *< 0.05; **, *P *< 0.01; ***, *P *< 0.001. Click here for file

Additional file 3 **TIG1 and GRK5 siRNAs increased PGE2-stimulated nuclear β-catenin accumulation in TIG1A-expressing cells**. Control or TIG1A stable cells were transfected with the indicated siRNA and then incubated with 5 nM MFP for 24 h. Cells were serum starved for 16 h and then stimulated with 10 nM PGE2 for 30 min. MFP was present during serum starvation and PGE2 treatment. β-catenin localization (green) and nuclei (blue) were analyzed using a laser scanning confocal microscope. Bars, 10 μm. Arrows indicate cells expressing nuclear β-catenin. Click here for file

Additional file 4 **Effects of PGE2 on nuclear β-catenin localization in control siRNA transfected TIG1A-expressing cells**. TIG1A stable cells were transfected with control siRNA and then incubated with 5 nM MFP for 24 h, serum starved for 16 h, and treated with or without 10 nM PGE2 for 30 min. MFP was present during serum starvation and PGE2 treatment. Nuclear and cytosolic fractions were prepared, and subcellular distribution of nuclear and cytosolic β-catenin was determined by Western blot analysis. Normalization in the levels of nuclear and cytosolic β-catenin was described in the Materials and Methods, and relative levels of nuclear β-catenin in cells without PGE2-treatment was designated as 1.0. Click here for file
