# Supplementary figures and images for: Tazarotene-induced gene 1 inhibits prostaglandin E2-stimulated HCT116 colon cancer cell growth
Source: J Biomed Sci. 2011 Nov 30;18(1):88. doi: 10.1186/1423-0127-18-88 (PMC3247857; doi:10.1186/1423-0127-18-88)

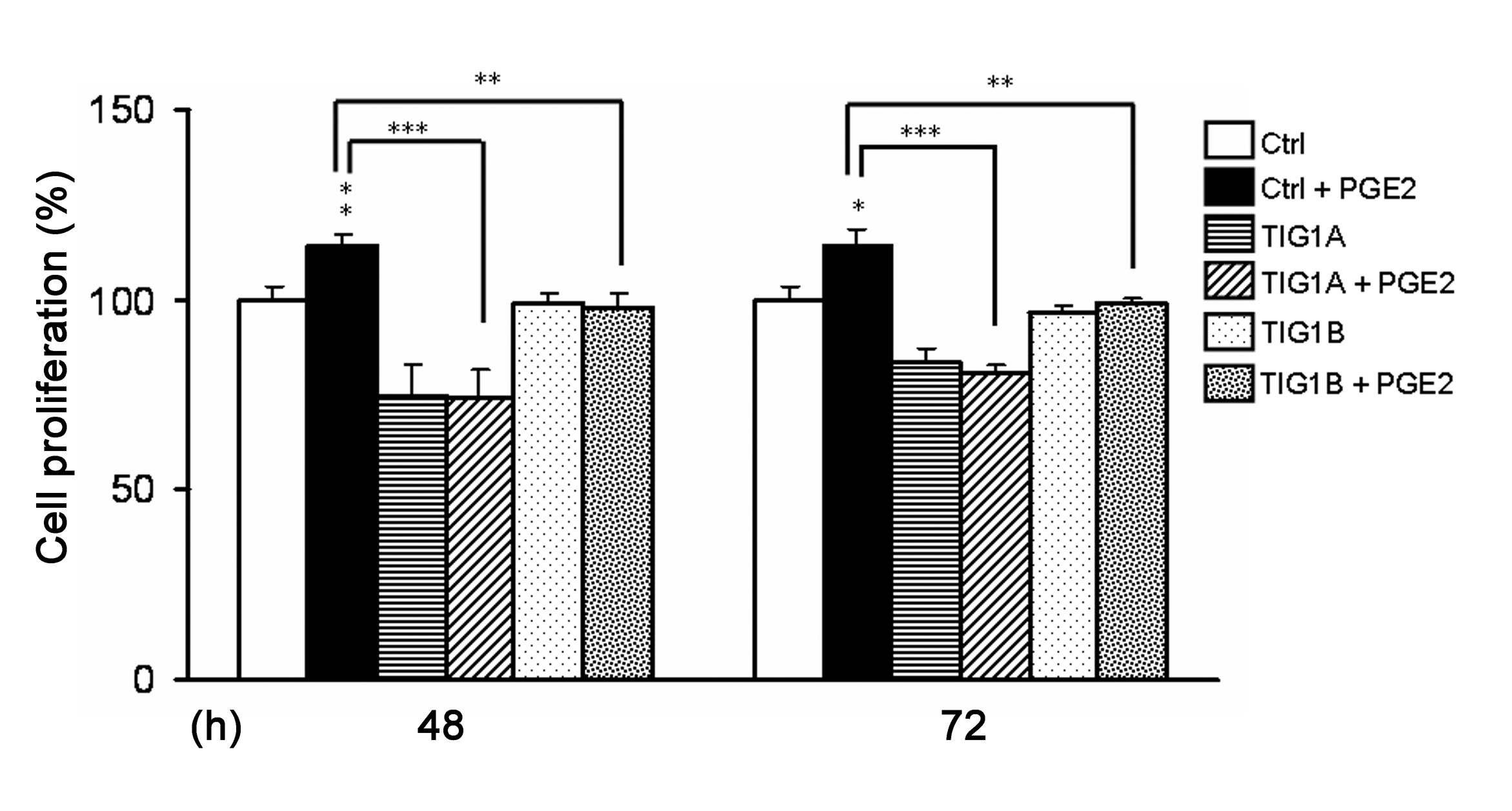

Supplement: Additional file 1 — Effects of TIG1A and TIG1B expression on PGE2-induced SW620 cell growth. Control, TIG1A and TIG1B inducible stable cells established from SW620 colon cancer cells using the GeneSwitch system [11] were plated overnight, serum starved for 16 h and then incubated in serum- free medium in the absence or presence of PGE2 (10 nM) for 48 to 72 h. MFP was present during serum starvation and PGE2 addition. Cell growth was determined using the WST-1 cell proliferation assay. *, P < 0.05. [file 1423-0127-18-88-S1.TIFF]

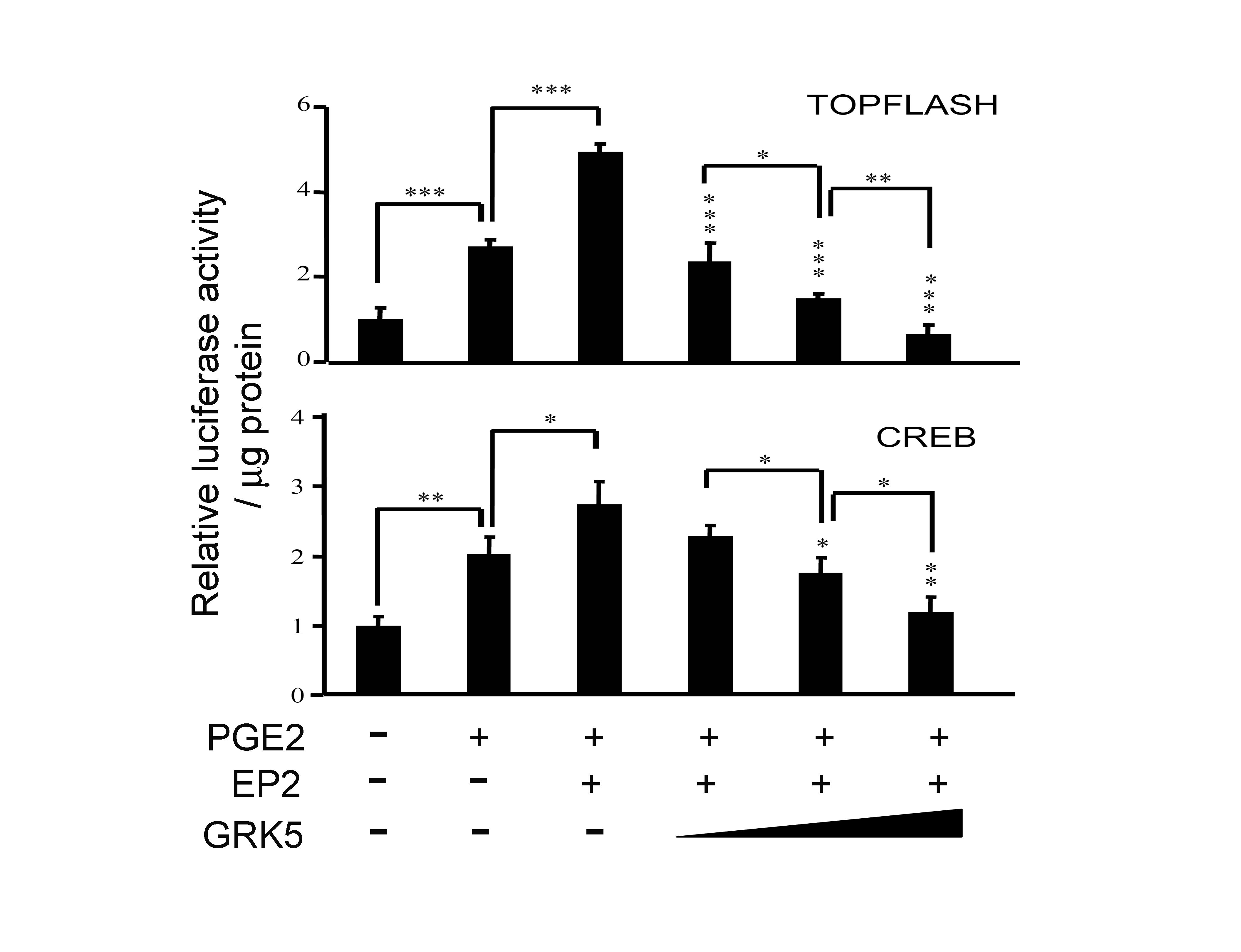

Supplement: Additional file 2 — GRK5 suppressed PGE2-stimulated TOPFLESH and CREB reporter activities in EP2-transfected HCT116 cells. HCT116 cells were transiently transfected with 250 ng of TOPFLASH or CREB reporter plasmid along with 100 ng of indicated control vector or EP2 TrucClone™ cDNA (PTGER2, OriGene Technologies, Inc. Rockville, MD, USA) and 50 to 150 ng of GRK5 expression vectors for 24 h. Cells were serum starved for 16 h and then incubated with or without 10 nM PGE2 for 24 h. Reporter activities were measured as described in the Materials and Methods. Representative results were expressed as means ± SD from triplicate samples. *, P < 0.05; **, P < 0.01; ***, P < 0.001. [file 1423-0127-18-88-S2.TIFF]

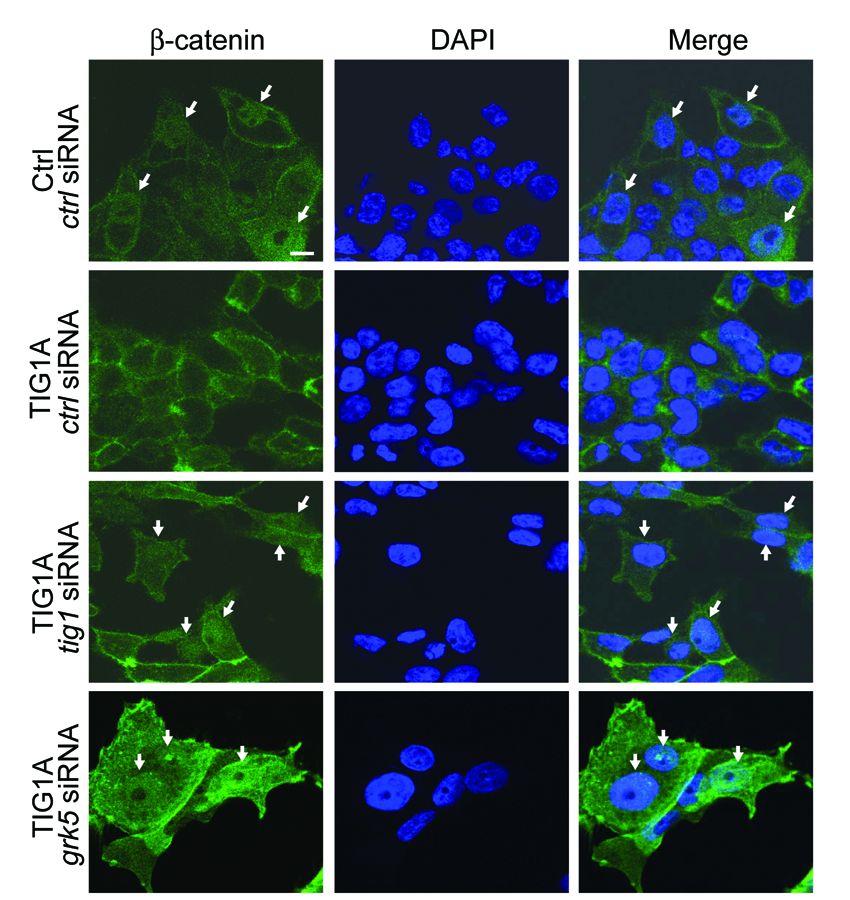

Supplement: Additional file 3 — TIG1 and GRK5 siRNAs increased PGE2-stimulated nuclear β-catenin accumulation in TIG1A-expressing cells. Control or TIG1A stable cells were transfected with the indicated siRNA and then incubated with 5 nM MFP for 24 h. Cells were serum starved for 16 h and then stimulated with 10 nM PGE2 for 30 min. MFP was present during serum starvation and PGE2 treatment. β-catenin localization (green) and nuclei (blue) were analyzed using a laser scanning confocal microscope. Bars, 10 μm. Arrows indicate cells expressing nuclear β-catenin. [file 1423-0127-18-88-S3.TIFF]

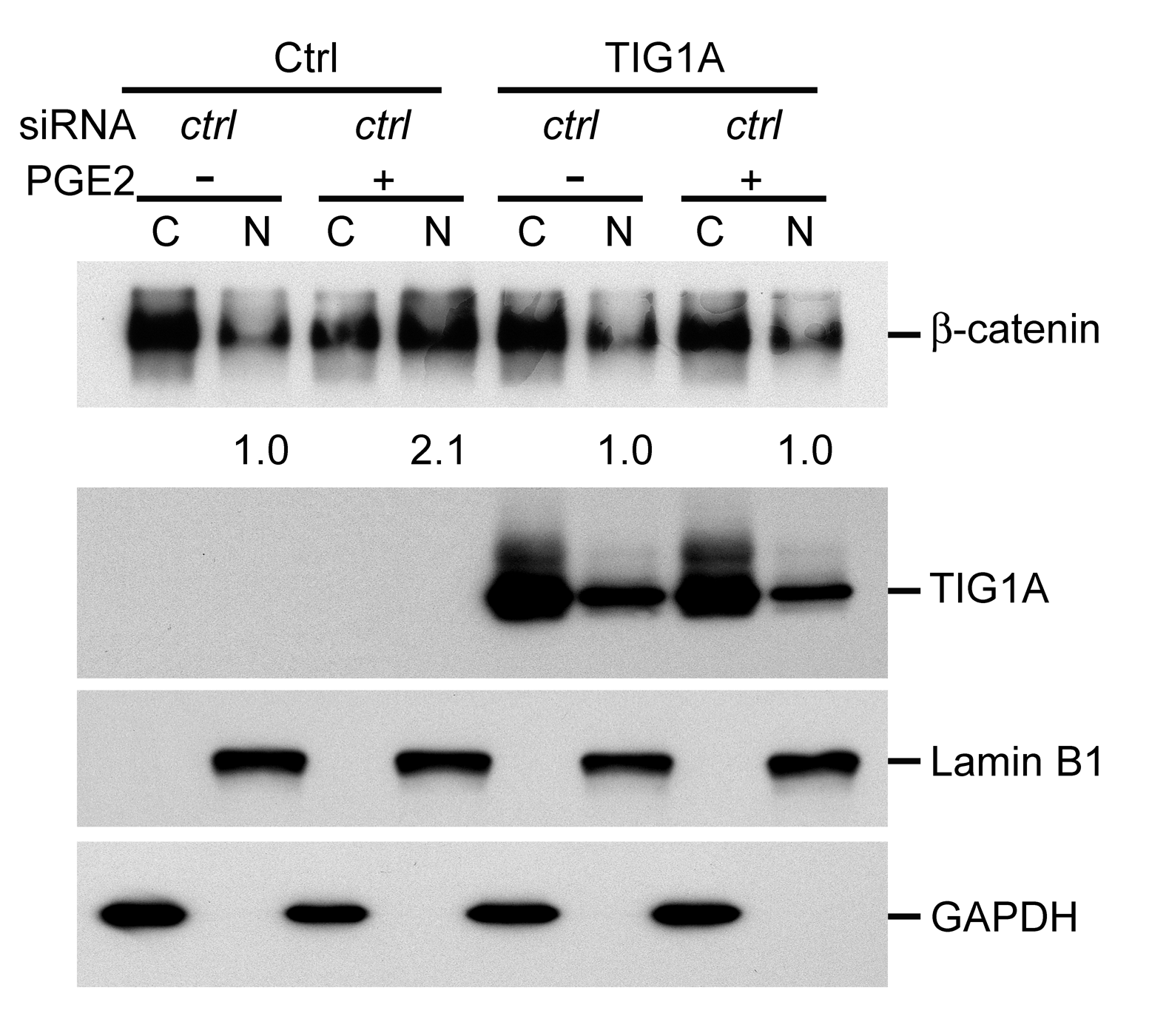

Supplement: Additional file 4 — Effects of PGE2 on nuclear β-catenin localization in control siRNA transfected TIG1A-expressing cells. TIG1A stable cells were transfected with control siRNA and then incubated with 5 nM MFP for 24 h, serum starved for 16 h, and treated with or without 10 nM PGE2 for 30 min. MFP was present during serum starvation and PGE2 treatment. Nuclear and cytosolic fractions were prepared, and subcellular distribution of nuclear and cytosolic β-catenin was determined by Western blot analysis. Normalization in the levels of nuclear and cytosolic β-catenin was described in the Materials and Methods, and relative levels of nuclear β-catenin in cells without PGE2-treatment was designated as 1.0. [file 1423-0127-18-88-S4.TIFF]
